# Estimating true prevalence of *Schistosoma mansoni* from population summary measures based on the Kato-Katz diagnostic technique

**DOI:** 10.1371/journal.pntd.0009310

**Published:** 2021-04-05

**Authors:** Oliver Bärenbold, Amadou Garba, Daniel G. Colley, Fiona M. Fleming, Rufin K. Assaré, Edridah M. Tukahebwa, Biruck Kebede, Jean T. Coulibaly, Eliézer K. N’Goran, Louis-Albert Tchuem Tchuenté, Pauline Mwinzi, Jürg Utzinger, Penelope Vounatsou

**Affiliations:** 1 Swiss Tropical and Public Health Institute, Basel, Switzerland; 2 University of Basel, Basel, Switzerland; 3 Department of Control of Neglected Tropical Diseases, World Health Organization, Geneva, Switzerland; 4 Schistosomiasis Consortium for Operational Research and Evaluation (SCORE), Center for Tropical and Emerging Global Diseases, University of Georgia, Athens, Georgia, United States of America; 5 Department of Microbiology, University of Georgia, Athens, Georgia, United States of America; 6 Schistosomiasis Control Initiative, Imperial College, London, United Kingdom; 7 Centre Suisse de Recherches Scientifiques en Côte d’Ivoire, Abidjan, Côte d’Ivoire; 8 Unité de Formation et de Recherche Biosciences, Université Félix Houphouët-Boigny, Abidjan, Côte d’Ivoire; 9 Vector Control Division, Ministry of Health, Kampala, Uganda; 10 Ministry of Health, Addis Ababa, Ethiopia; 11 Laboratory of Parasitology and Ecology, University of Yaoundé I, Yaoundé, Cameroon; 12 Centre for Schistosomiasis and Parasitology, Yaoundé, Cameroon; 13 Centre for Global Health Research, Kenya Medical Research Institute, Nairobi, Kenya; University of Glasgow School of Life Sciences, UNITED KINGDOM

## Abstract

**Background:**

The prevalence of *Schistosoma mansoni* infection is usually assessed by the Kato-Katz diagnostic technique. However, Kato-Katz thick smears have low sensitivity, especially for light infections. Egg count models fitted on individual level data can adjust for the infection intensity-dependent sensitivity and estimate the ‘true’ prevalence in a population. However, application of these models is complex and there is a need for adjustments that can be done without modeling expertise. This study provides estimates of the ‘true’ *S. mansoni* prevalence from population summary measures of observed prevalence and infection intensity using extensive simulations parametrized with data from different settings in sub-Saharan Africa.

**Methodology:**

An individual-level egg count model was applied to Kato-Katz data to determine the *S. mansoni* infection intensity-dependent sensitivity for various sampling schemes. Observations in populations with varying forces of transmission were simulated, using standard assumptions about the distribution of worms and their mating behavior. Summary measures such as the geometric mean infection, arithmetic mean infection, and the observed prevalence of the simulations were calculated, and parametric statistical models fitted to the summary measures for each sampling scheme. For validation, the simulation-based estimates are compared with an observational dataset not used to inform the simulation.

**Principal findings:**

Overall, the sensitivity of Kato-Katz in a population varies according to the mean infection intensity. Using a parametric model, which takes into account different sampling schemes varying from single Kato-Katz to triplicate slides over three days, both geometric and arithmetic mean infection intensities improve estimation of sensitivity. The relation between observed and ‘true’ prevalence is remarkably linear and triplicate slides per day on three consecutive days ensure close to perfect sensitivity.

**Conclusions/significance:**

Estimation of ‘true’ *S. mansoni* prevalence is improved when taking into account geometric or arithmetic mean infection intensity in a population. We supply parametric functions and corresponding estimates of their parameters to calculate the ‘true’ prevalence for sampling schemes up to 3 days with triplicate Kato-Katz thick smears per day that allow estimation of the ‘true’ prevalence.

## Author summary

The World Health Organization (WHO) recommends the Kato-Katz diagnostic method, i.e., counting eggs in a thick-smear of stool using light microscopy, for estimation of *Schistosoma mansoni* infection prevalence. While the diagnostic specificity of Kato-Katz is very high, the sensitivity varies strongly with infection intensity and the number of samples collected and thick smears per sample tested. Therefore, the performance of Kato-Katz in a population depends on the distribution of infections in the population and individual-level data are needed to determine the ‘true’ prevalence of infection. However, modeling capacity to determine ‘true’ prevalence from individual-level data is often not available to program managers. In this study, we therefore provide simple equations to estimate the ‘true’ prevalence and associated uncertainty from observed prevalence and arithmetic or geometric mean infection intensity for a variety of common sampling schemes. We find that by including information about the mean infection intensity in a population the estimation of ‘true’ prevalence can be improved compared to assuming a constant value for the diagnostic sensitivity and supply parameters and functions to calculate the ‘true’ prevalence of infection.

## Introduction

Schistosomiasis is a neglected tropical disease caused by infection with parasitic flatworms that have a complex life cycle, including freshwater snails as an intermediate host [1]. The most recent Global Burden of Disease Study gives an estimate of 1.43 million disability-adjusted life years primarily among the school-age population of sub-Saharan Africa [2]. The World Health Organization (WHO) has set the goal of eliminating morbidity due to schistosomiasis by 2025, using preventive chemotherapy in regions that surpass given thresholds for the prevalence of the disease [3].

*Schistosoma mansoni* is most commonly diagnosed using the Kato-Katz technique, a parasitologic method that relies on the detection of eggs in a thick smear of stool (41.7 mg), examined under a light microscope by experienced laboratory technicians [4]. However, the diagnostic sensitivity of the technique is low; in the order of 50%, for single or duplicate thick smears obtained from one stool sample. The sensitivity is influenced by the sampling scheme, i.e., the number of days on which sampling is done and the number of slides per sample [5–7]. The sensitivity increases with the intensity of infection (proxied by the number of eggs in the stool of an individual). However, for very light infections even sampling on many days does not lead to a sensitivity close to 100% and therefore prevalence remains underestimated [8–10].

The WHO defines intervention thresholds for preventive chemotherapy with praziquantel based on observed prevalence, while not taking into account infection intensity and sensitivity of the diagnostic method [11]. Using unadjusted observed prevalence from widely different settings is likely to introduce considerable bias. To administer preventive chemotherapy effectively and to track progress over time, historic data generated under various sampling schemes need to be integrated in a coherent model to reduce risk of bias.

Recent studies have shown that the underlying ‘true’ prevalence can be estimated effectively when individual-level count data are utilized [8, 12, 13]. Alternatively, latent class models can be employed on binary data to adjust for the imperfect sensitivity of Kato-Katz thick smears and improve on the most basic approach of assuming the combined results of multiple Kato-Katz tests constitute a ‘gold’ standard. However, modeling capacity to determine ‘true’ prevalence from individual-level count data or latent class models is often not available to program managers. Population summary measures like observed prevalence, arithmetic or geometric mean on the other hand are simple to calculate. Hence, there is a need to estimate the ‘true’ prevalence from these population summary measures only. Previously, de Vlas et al. have developed a pocket chart to achieve the same goal but without taking into account sampling schemes and associated uncertainty in the estimates [14]. The aim of this study is to provide equations for simple calculation of the ‘true’ prevalence and associated uncertainty for various sampling schemes from population summary measures.

We carried out a simulation study to determine the relation between the ‘true’ prevalence—defined as the proportion of individuals with at least one worm-pair—and the observed prevalence as well as summary measures for infection intensity. The model is based on a worm-mating model and takes into account variations in egg excretion that influence the diagnostic process. Our modeling results are validated using national survey data from Uganda. Taken together, we propose a simple way for disease control managers to calculate ‘true’ prevalence from observed prevalence, while taking into account infection intensity. Additionally, we supply informative priors to be used in *S. mansoni* modeling studies, such as latent class analysis using a Bayesian formulation.

## Methods

### Ethics statement

All data included in this study have been published elsewhere [15–23]. Ethics approval, written informed consent procedures, and treatment of infected individuals are given in the aforementioned studies from which the data have been extracted.

### Data

In this study, we used a suite of 20 datasets with Kato-Katz thick smear results available for between two and three thick smears on each of 2 or 3 days. The data are summarized in Table 1. The data originate from five countries in sub-Saharan Africa; namely Cameroon, Côte d’Ivoire, Ethiopia, Kenya, and Uganda. The observed prevalence of *S. mansoni* ranged from 3.8% to 91.7%, mean infection intensity from 37 eggs per gram of stool (EPG) to 525 EPG, and the geometric mean from 8.5 EPG to 248 EPG. Two or three Kato-Katz thick smears were prepared from stool samples on 2 or 3 days from between 100 and 1,845 individuals. The data are used in the egg count model described below to infer on the infection intensity-dependent sensitivity of the Kato-Katz technique.

**Table 1:** Summary of the survey data used to parametrize the current simulation study.

Country	Location	Age range(years)	$n_{d}^{1}$	$n_{s}^{2}$	N_KK_	Prevalence (%)	arith. *μ* (EPG)	geom. *μ* (EPG)	Source
Cameroon	Makanene	6-16	3	3	251	71.7	161	43.3	[17]
Cameroon	Njobe	8-16	3	3	245	63.3	173	27.5	[17]
Cameroon	Yaonde	7-14	3	3	233	27.9	235	40.9	[17]
Côte d’Ivoire	12 villages in the western part	9-12	2	3	695	6.5	72	22.0	[23]
Côte d’Ivoire	1	0.2-5.5	2	2	109	25.7	90	37.0	[19]
Côte d’Ivoire	2	0.2-5.5	2	2	133	21.1	122	30.8	[19]
Côte d’Ivoire	1	8-12	3	3	170	91.7	525	248	[15]
Côte d’Ivoire	2	8-12	3	3	130	53.1	116	36.8	[15]
Côte d’Ivoire	3	8-12	3	3	146	32.9	50	8.5	[15]
Ethiopia	Harbu	8-12	3	2	300	57.0	69	31.0	[20]
Ethiopia	Jiga	8-12	3	2	320	49.4	153	70.9	[20]
Kenya	Usoma	1-15	3	2	1,845	22.1	106	32.1	[16]
Uganda	1	7-13	3	2	100	55.0	240	34.2	[21]
Uganda	2	7-13	3	2	100	54.0	122	33.3	[21]
Uganda	3	7-13	3	2	100	31.0	37	19.8	[21]
Uganda	4	7-13	3	2	100	35.0	247	58.0	[21]
Uganda	5	7-13	3	2	100	12.0	58	28.4	[21]
Uganda	Base		3	2	775	6.3	48	22.0	
Uganda	F1		3	2	659	4.2	68	33.5	
Uganda	Mapping		3	2	711	3.8	182	26.9	

^1^ *n*_*d*_ is the number of stool specimens taken on different days

^2^ *n*_*s*_ is the number of Kato-Katz thick smears prepared from each stool specimen

arith., arithmetic; EPG, eggs per gram of stool; geom., geometric

Additionally, data from a national survey carried out in 2016 in Uganda, primarily along the coast of Lake Victoria were used for validation purposes. The data can be found in S1 Table. There were a total of 146 locations of which we purposefully selected those 34 schools for validation where the observed prevalence was above 10% after examination of duplicate Kato-Katz thick smears of a single stool sample. There were 33 schools with 48-56 children and one school with 104 children. The observed *S. mansoni* prevalence ranged from 12% to 87.5% with 26 schools having a prevalence below 50%. The arithmetic mean ranges from 9.8 EPG to 820 EPG, and the geometric mean from 3.8 EPG to 233 EPG.

### Procedures

We fitted the real data across Africa on an egg count model estimating the sensitivity of the Kato-Katz technique as a function of infection intensity. We employed the egg count model from our earlier work and extended it in this study [8]. We simulated worm burden and observed data at individual level in a hypothetical population using the transmission model explained below, and taking into account the estimated sensitivity of Kato-Katz. A statistical model was fitted on the simulated data relating diagnostic sensitivity with ‘observed’ population mean egg intensity and prevalence. The model was used to predict ‘true’ prevalence from observed prevalence data by Kato-Katz when the ‘observed’ arithmetic or geometric mean is available, taking into account diagnostic sensitivity across sampling schemes varying from a single to duplicate slides per stool sample from 1 to 3 days. Details on the modeling, simulation approach, and validation methods are given below, while a schematic of how the different model parts are connected is in the supplementary S1 Fig.

#### Modeling diagnostic sensitivity of Kato-Katz

To model the data generating process, we extended our Kato-Katz egg count model described elsewhere [8]. In this model, the infected population *j* with *S. mansoni* prevalence *p*_*j*_ is characterized by an arithmetic mean egg intensity *μ*_*j*_, while the infection intensity λ_*ji*_ of individual *i* is assumed to follow a shifted gamma distribution with a rate parameter *α*_*j*_ and a shift *μ*_*min*_ corresponding to the lowest possible infection with one worm pair.

$$\begin{array}{c} \lambda_{ji}∼\text{Gamma}\left(\left(\mu_{j}-\mu_{min}\right)\cdot \alpha_{j},\alpha_{j}\right) \end{array}$$ (1)

To determine the sensitivity of Kato-Katz, we modified our existing model to take into account (i) the day-to-day variation of egg output of an individual and (ii) the heterogeneous distribution of eggs within a single Kato-Katz thick smear. In particular, we modeled the variation by a log-normal distribution, that is the egg output of individual *i* in population *j* on day *d*, log(λ_*jid*_) = log(λ_*ji*_ + *μ*_*min*_) + *ϵ*_*jid*_ where $\epsilon_{jid}∼N\left(-\sigma_{j}^{2}/2,\sigma_{j}\right)$. We assumed that the observed Kato-Katz egg count data $Y_{jids}^{KK}$ show a negative binomial distribution $Y_{jids}^{KK}∼NB\left(\lambda_{jid},\gamma_{j}\right)$. The parameter *γ*_*j*_ captures the over-dispersion in the egg count data from a single individual in population *j* [7]. *γ*_*j*_ and *σ*_*j*_ are allowed to vary between studies around a common mean, $\gamma_{j}∼LN\left(\text{log}\left(\gamma \right)-\sigma_{\gamma }^{2}/2,\sigma_{\gamma }\right)$ and $\sigma_{j}∼LN\left(\text{log}\left(\sigma \right)-\sigma_{\sigma }^{2}/2,\sigma_{\sigma }\right)$.

False-negative results are included in the model as repeated zero measurements, thus, the sensitivity for a single Kato-Katz thick smear reading of an individual with egg-density λ_*jid*_ becomes

$$\begin{array}{c} s_{jid}^{KK}=1-\text{NB}\left(0,\lambda_{jid},\gamma_{j}\right)=1-{\left(\frac{\gamma_{j}}{\lambda_{jid}+\gamma_{j}}\right)}^{\gamma_{j}} \end{array}$$ (2)

For the non-infected individuals, the mean egg-density λ_*ji*_, and hence, the counts $Y_{jids}^{KK}$ are set to zero.

The infection intensity of one pair of worms *μ*_*min*_ is fixed to the expected average egg output of a pair of worms, which is in the order of 100 eggs, multiplied by the ratio between the weight of a Kato-Katz thick smear and the average daily production of feces, which corresponds to about 0.05 eggs per slide (EPS) or 1.2 EPG for *S. mansoni* [24]. The unit of EPS is used for modeling because it is the unit of measurement while results are usually communicated in EPG. All models were formulated using a Bayesian framework of inference and fitted by Markov chain Monte Carlo (MCMC) simulation in Stan version 2.16.2 (Stan Development Team; mc-stan.org) [25]. Priors were chosen as a normal distribution with mean 0.5 and standard deviation (SD) 0.5 for *α*_*j*_, a gamma distribution with mean 25 and SD 125 for *μ*_*j*_, truncated normal distribution with mean parameter 0 and SD parameter 3 for *σ*, truncated normal distribution with mean parameter 0 and SD parameter 1 for *σ*_*σ*_, truncated normal distribution with mean parameter 1 and SD parameter 3 for *γ*, and a truncated normal distribution with mean parameter 0 and SD parameter 1 for *σ*_*γ*_. Semi-informative priors on variance parameters and mean infection intensity were used to limit estimates in datasets with few positives to realistic values. The results were not sensitive to the exact choice of priors. Model code can be found in S1 File.

#### Simulating worm burden and observed *S. mansoni* infection data

We assumed that the distribution of worms in a population can be simulated using a negative binomial distribution, which is a commonly employed assumption in transmission models for schistosomiasis [24]. Using the model for the worm mating process by May and Woolhouse (1993), the distribution of worm-pairs is determined [26, 27]. The distribution is defined by the mean number of worms $\mu_{j}^{w}$ in the population *j*, the aggregation parameter of the negative binomial distribution of worms *k*_*j*_ in the population *j*, *q*_*w*_ the proportion of female worms, and the mean number of eggs per worm-pair *n*_*w*_ set to 0.2 EPS [28]. The aggregation parameters *k*_*j*_ are assumed to be related between different populations *j* and distributed log-normally around a common mean *k*_*j*_ ∼ *LN*(log(*k*_0_) − *δ*^2^/2, *δ*), while the $\mu_{j}^{w}$ are independent and related to the force of transmission. Under the assumption of a negative binomial distribution, the prevalence—defined as the individuals with zero worm pairs—can be calculated as follows with *p*_*j*_ being the prevalence.

$$\begin{array}{c} p_{j}=1-{\left(1+\frac{q_{w}\mu_{j}^{w}}{k_{j}}\right)}^{-k_{j}}-{\left(1+\frac{\left(1-q_{w}\right)\mu_{j}^{w}}{k_{j}}\right)}^{-k_{j}}+{\left(1+\frac{\mu_{j}^{w}}{k_{j}}\right)}^{-k_{j}} \end{array}$$ (3)

We simulated individual level data for 9,000 hypothetical populations according to the egg count model presented above, using the posterior of the fit to the datasets above as priors for *σ* and *γ*. Population size *N* was chosen as 30 or 50, which are two common population sizes for schistosomiasis mapping studies, and as 5,000 to exclude influence of sampling error. We varied the mean intensity of infected individuals from an average of 10 to 400 worms per individual in 15 steps on a logarithmic scale, which covers a wide range of possible scenarios. We simulated 200 populations for each combination using a new draw from the joint posterior distribution for each population. Sampling schemes considered for Kato-Katz were *d*1*s*1 (1 day and 1 slide per sample), *d*1*s*2 (1 day and 2 slides), *d*2*s*1 (2 days and 1 slide), *d*2*s*2, *d*3*s*2, *d*3*s*3, where the number after *s* denotes the number of slides per day and the number after *d* the number of days where sampling was done.

For each simulated population and for each sampling scheme, we calculated four different summary measures for the mean (i.e., arithmetic and geometric mean for all individuals as well as for only the positive ones). The full arithmetic mean *μ*_*ja*_ of the population *j* is simply the mean egg count of all slides over all days and individuals. To calculate the full geometric mean *μ*_*jg*_, 1 has to be added to the counts to avoid taking the logarithm of zero, then the geometric mean can be calculated using the standard formula, and finally, 1 is subtracted again. The mean arithmetic, $\mu_{ja}^{+}$ and geometric $\mu_{jg}^{+}$ infection intensity in the positive population was calculated by

$$\begin{array}{c} \begin{array}{cc} \mu_{ja}^{+} & =\frac{\mu_{ja}}{p_{j}^{obs}}\;\text{and}\\ \mu_{jg}^{+} & ={\left(\mu_{jg}+1\right)}^{1/p_{j}^{obs}}-1 \end{array} \end{array}$$ (4)

where $p_{j}^{obs}$ is the observed prevalence. Because the full mean and the mean of positives are related directly via the prevalence, having one of the two means is sufficient to calculate the other.

The individual level sensitivity of the Kato-Katz technique was obtained using Eq 2 based on the true infection intensity of individual *i*, in the simulated population *j*, on day *d*. The mean of all sensitivities in a population was calculated to determine the sample sensitivity, i.e., $s_{ji}^{KK,ds}=1-\prod_{D=1}^{d}{\left(\frac{\gamma_{j}}{\lambda_{jiD}+\gamma_{j}}\right)}^{s\cdot \gamma_{j}}$. Simulation code can be found in S2 File.

### Relating ‘true’ prevalence to observed prevalence and mean infection intensity

We fitted a parametric statistical model on simulated data to estimate population-level sensitivity under the assumptions that (i) the mean sensitivity can be expressed as a function of the infection intensity and (ii) the sensitivity values are described by a beta distribution. *z* represents either a linear transformation of the geometric mean *μ*_*g*_/8 or the arithmetic mean *μ*_*a*_/25 of the full population. Division by 8 and 25 for geometric and arithmetic mean, respectively, ensures that the range of values for *z* is mostly within 0 and 1 optimising computation using MCMC for this model.

$$\begin{array}{c} \begin{array}{cc} s_{n}^{KK,ds} & ∼Beta\left(\alpha_{n},\beta_{n}\right),\;\alpha_{n}=\frac{m_{n}}{\nu_{n}},\;\beta_{n}=\frac{1-m_{n}}{\nu_{n}}\\ \text{logit}\;m_{n} & =a_{0}+a_{1}\cdot z_{n}^{1/a_{2}},\;\text{log}\nu_{n}=b_{0}+b_{1}\cdot \text{log}\left(z_{n}\right) \end{array} \end{array}$$ (5)

*m*_*n*_ is the estimated sensitivity for a given value *z*_*n*_, where *a*_0_ determines the sensitivity at low values for *z*, *a*_1_ the increase with *z*, and *a*_2_ the shape of the curve. *ν*_*n*_ determines the variance and is modeled using a linear model in log(*z*_*n*_) with parameters *b*_0_ and *b*_1_.

We also determined the relation between observed $p_{n}^{obs}$ and ‘true’ *p*_*n*_ prevalence, using the following model ensuring a linear relation between $p_{n}^{obs}$ and *p*_*n*_.

$$\begin{array}{c} \begin{array}{cc} p_{n}^{KK,ds} & ∼Beta\left(\alpha_{n},\beta_{n}\right),\;\alpha_{n}=\frac{m_{n}}{\nu_{n}},\;\beta_{n}=\frac{1-m_{n}}{\nu_{n}}\\ m_{n} & =\left(2\;{\text{logit}}^{-1}\left(a_{1}p_{n}^{obs}\right)-1\right)\left(1-a_{0}\right)+a_{0}),\;\nu_{n}=b_{0}+b_{1}\cdot p_{n}^{obs} \end{array} \end{array}$$ (6)

*m*_*n*_ is the estimated ‘true’ prevalence *p*_*n*_ for a given observed prevalence $p_{n}^{obs}$, where *a*_0_ determines the true prevalence at very low observed prevalence, *a*_1_ describes the increase of the ‘true’ prevalence with increasing observed prevalence. *ν*_*n*_ determines the variance and is modeled using the two parameters *b*_0_ and *b*_1_. Model code can be found in S3–S5 Files.

### Validation

We validated the estimated relation between the population sensitivity and the mean infection intensity using survey data from Uganda described in the data section. We estimated sensitivity and ‘true’ prevalence using the egg count model presented previously and calculated summary measures according to the definitions given above. Validation was done by visual comparison between simulated populations and the estimates from the schools in the validation dataset. Validation model code can be found in supplementary file S6 File.

## Results

### Kato-Katz day-to-day and slide-to-slide variations

Estimates of the day-to-day and slide-to-slide variation in egg counts of the Kato-Katz diagnostic technique obtained from 20 different datasets collected in five countries of sub-Saharan Africa are shown in Table 1. The parameter that catches the day-to-day variation, *σ*, was estimated at 1.16 (95% Bayesian credible interval (BCI) 1.03–1.23), and the parameter of the negative binomial count distribution that captures the slide-to-slide variation, *δ*, at 6.14 (95% BCI 4.36–8.50) (posterior mean and 95% BCI in brackets).

The infection-intensity dependent sensitivity for six different sampling schemes, including one or two slides per stool sample from 1 to 3 days calculated using the estimates for *σ* and *δ* is shown in S2 Fig. Above 200 EPG, even a single slide achieves a sensitivity of 90%, while at 10 EPG, not even three samples with two slides each reach a sensitivity of 75%. Two slides from different days show consistently higher sensitivity than duplicate Kato-Katz thick smears prepared from the same day due to the stronger variations between days than slides.

The estimate of the infection intensity of a single pair of worms is around 0.2 EPS or 4.8 EPG. However, this estimate is uncertain and the true value might well be 1 EPG. Thus, the minimum sensitivity of Kato-Katz for three stool samples on consecutive days with two slides each might be as low as 25%, as shown also in S3 Fig.

### Simulation of populations

**Fig 1:**
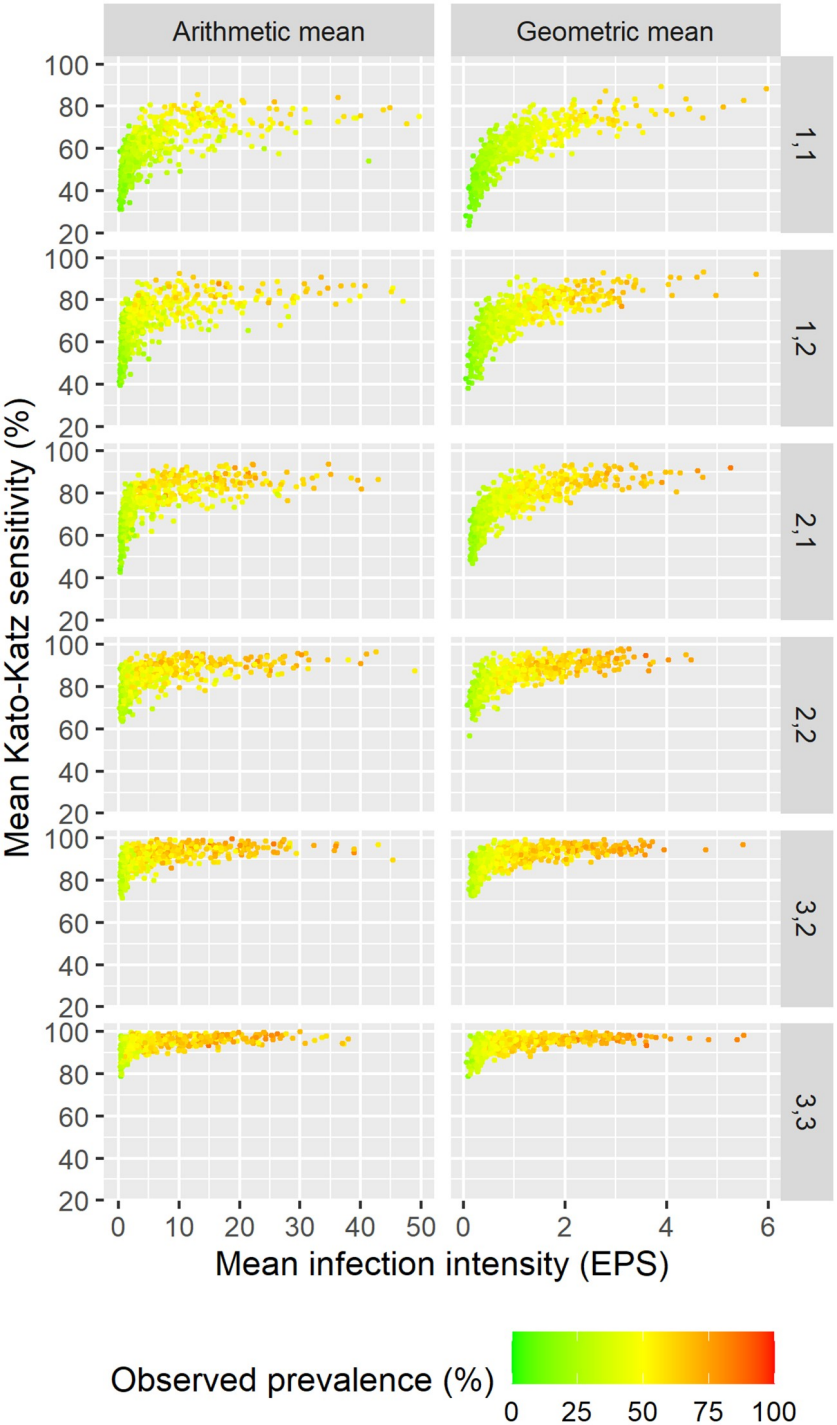
Overall sensitivity of Kato-Katz in relation to geometric and arithmetic mean of total population for six different sampling schemes in simulations with 50 individuals per location. The sampling scheme is denoted on the right side with the first number referring to the number of stool samples and the second to the number of slides per stool sample. Observed prevalence is shown in color and EPS refers to eggs-per-slide.

Fig 1 shows total sensitivity in a population of 50 individuals, that is the mean sensitivity across all positive individuals, in relation to arithmetic and geometric mean infection intensity of the total population for six different sampling schemes. There is a clear relation between infection intensity and sensitivity for both geometric and arithmetic mean, while variations for the former are smaller. Sampling on three days with three slides leads to high overall sensitivity over 80% for even very low mean infection intensity. Two slides on a single day, the sampling scheme recommended by WHO, shows a sensitivity between 40% and 80%, depending on infection intensity.

The relation between the mean of only the positive individuals and the total sensitivity is shown in S4 Fig. The relation between infection intensity and sensitivity is still visible in both arithmetic and geometric mean of the positives but variations are much larger. Estimation of mean of the positives only is based on a considerably lower sample size than mean of the total population and hence, carries a larger uncertainty. Hence, we fitted the statistical model to the means of the total population only to profit from the lower uncertainty.

**Fig 2:**
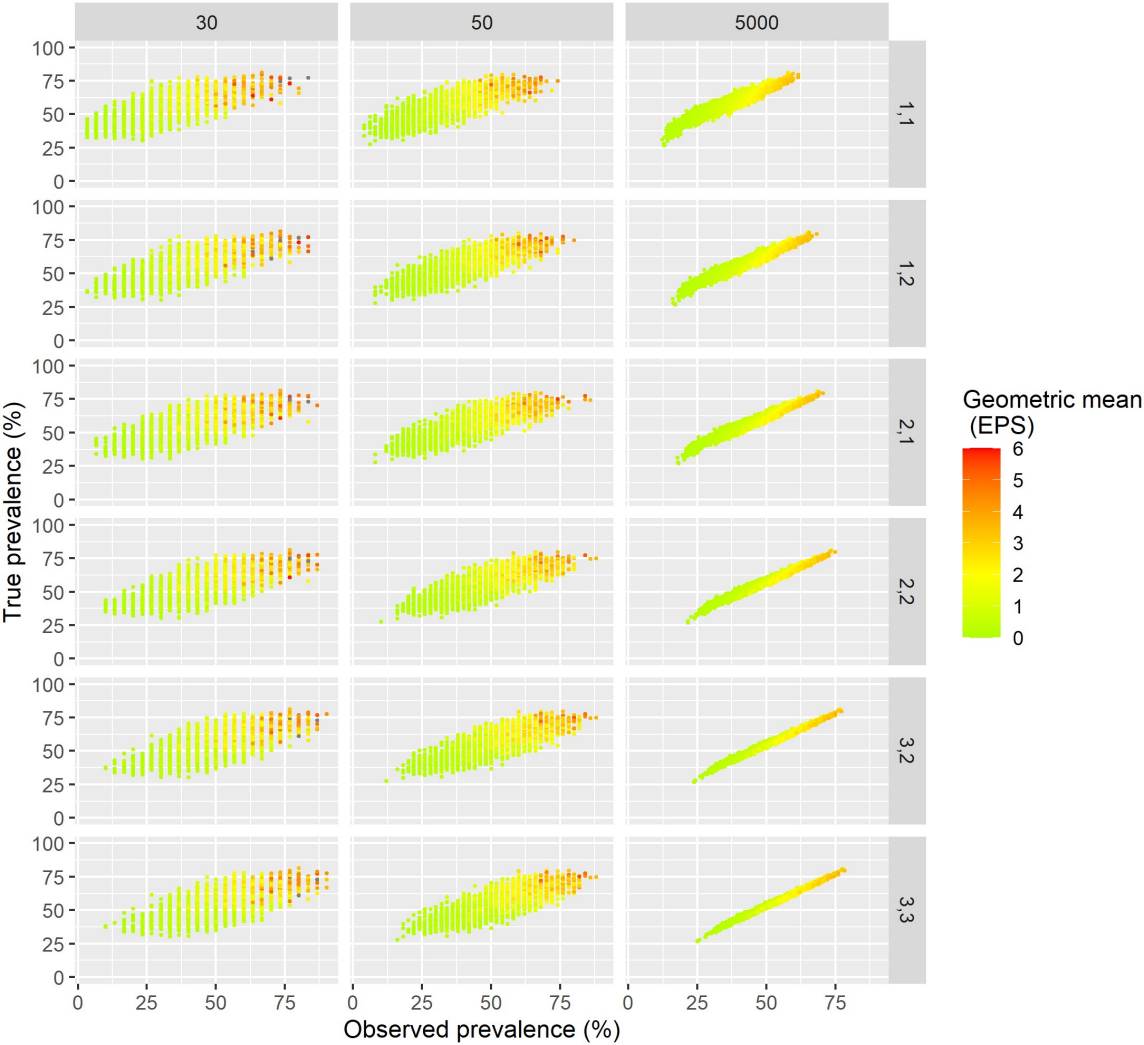
Relation between observed prevalence and ‘true’ prevalence for sample sizes of 30, 50, and 5,000 with geometric mean of the total population in color. The sampling scheme applied is denoted on the right hand side where the first number is the number of stool samples and the second the number of slides per day. The geometric mean infection intensity is shown in color and EPS refers to eggs-per-slide.

The relation between observed prevalence and ‘true’ prevalence is presented in Fig 2 for sample sizes of 30, 50, and 5,000 and each of the six sampling schemes. The relation appears fairly linear. As expected, the larger the sample size, the smaller the uncertainty. The observed prevalence is almost equivalent to ‘true’ prevalence for samples based on 3 days with triplicate slides each, confirming that sensitivity is very high for that sampling scheme. Uncertainty is considerably larger for lower sample sizes of 30 or 50 making the ‘true’ prevalence more difficult to estimate.

### Relation between observed summary measures and ‘true’ prevalence

Table 2 shows the posterior means for the parameters defining the relation between diagnostic sensitivity of different Kato-Katz sampling schemes and the geometric mean intensity of the total population. The mean estimate for the sensitivity can be calculated from the parameters using Eq 5 given the geometric mean infection intensity in EPS. *a*_0_, the parameter that describes the sensitivity for very low mean infection intensity increases steadily with larger sampling effort, indicating a higher sensitivity. The change is less pronounced in the fit for sample size 30. There is a slight reduction in *b*_1_, the parameter describing the change of the uncertainty in the sensitivity with infection intensity. S2 and S3 Tables present the parameter values of the fit between arithmetic mean infection intensity and sensitivity as well as observed prevalence and ‘true’ prevalence.

**Table 2:** Posterior estimates (mean and 95% BCI) of the parameters describing the relation between geometric mean of the total population and sensitivity for six sampling schemes and two sample. The mean estimate for the ‘true’ prevalence is calculated by $p=p^{obs}/{\text{logit}}^{-1}\left(a_{0}+a_{1}\cdot {\left(\mu_{g}\right)}^{1/a_{2}}\right)$ when the geometric mean infection intensity *μ*_*g*_ in eggs-per-slide (EPS) is known. The probability distribution of the ‘true’ prevalence is given by Beta(*α*,*β*), where *α* = *p*/*ν*, *β* = (1 − *p*)/*ν*, and *ν* = exp(*b*_0_ + *b*_1_log(*μ*_*g*_)).

Scheme	*a*_0_	*a*_1_	*a*_2_	*b*_0_	*b*_1_
N = 50					
11	-7.19 (-9.55–-5.36)	8.98 (7.19–11.31)	13.54 (10.28–17.73)	-4.54 (-4.69–-4.39)	-0.06 (-0.12–0.00)
12	-6.24 (-8.55–-4.39)	8.45 (6.63–10.73)	13.51 (10.01–17.92)	-4.45 (-4.60–-4.30)	-0.08 (-0.14–-0.02)
21	-6.38 (-8.89–-4.36)	8.86 (6.88–11.34)	13.92 (10.16–18.63)	-4.54 (-4.70–-4.40)	-0.09 (-0.15–-0.02)
22	-5.09 (-7.62–-3.09)	8.06 (6.10–10.57)	13.52 (9.57–18.59)	-4.62 (-4.77–-4.47)	-0.14 (-0.20–-0.08)
32	-4.12 (-6.61–-2.26)	7.57 (5.73–10.03)	13.29 (9.37–18.41)	-4.97 (-5.13–-4.82)	-0.25 (-0.31–-0.19)
33	-3.30 (-5.54–-1.40)	7.08 (5.22–9.28)	13.32 (9.06–18.30)	-5.3 (-5.45–-5.14)	-0.33 (-0.39–-0.27)
N = 30					
11	-6.03 (-8.03–-4.54)	7.86 (6.41–9.83)	11.41 (8.73–15.00)	-4.11 (-4.24–-3.97)	-0.07 (-0.12–-0.01)
12	-5.16 (-7.37–-3.50)	7.44 (5.82–9.61)	11.25 (8.14–15.36)	-3.99 (-4.13–-3.85)	-0.09 (-0.15–-0.04)
21	-5.41 (-7.68–-3.60)	7.93 (6.16–10.17)	12.03 (8.66–16.25)	-4.10 (-4.24–-3.96)	-0.10 (-0.16–-0.05)
22	-4.12 (-6.43–-2.29)	7.12 (5.34–9.39)	11.55 (7.93–16.11)	-4.15 (-4.29–-4.00)	-0.17 (-0.22–-0.11)
32	-3.62 (-6.04–-1.67)	7.05 (5.14–9.46)	12.42 (8.26–17.60)	-4.42 (-4.56–-4.27)	-0.22 (-0.28–-0.17)
33	-2.80 (-5.12–-0.96)	6.56 (4.75–8.83)	12.51 (8.25–17.72)	-4.76 (-4.9–-4.61)	-0.31 (-0.37–-0.25)

There is a good fit in the relation between the geometric mean infection intensity and the diagnostic sensitivity in a population (see also S5 Fig). The relation between the arithmetic mean infection intensity and the sensitivity (S6 Fig) is similar to the one determined for the geometric mean infection, but the uncertainty is slightly larger possibly because the arithmetic mean is influenced more by outliers. Taking three Kato-Katz thick smears from three stool samples achieves observed prevalence basically equivalent to the ‘true’ prevalence, while for duplicate Kato-Katz thick smears from one stool sample, the observed prevalence is only half of the ‘true’ prevalence. For example, an observed prevalence of 25% corresponds to a ‘true’ prevalence of 50%. The relation between observed and ‘true’ prevalence appears linear for each sampling scheme (S7 Fig). However, uncertainty is large for a sample size of only 50.

### Validation

**Fig 3:**
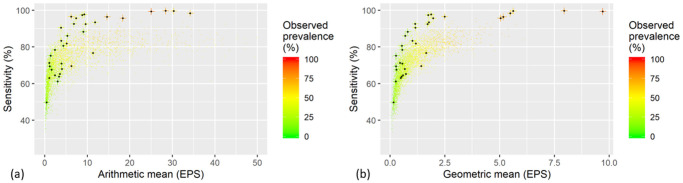
Estimated sensitivity in relation to infection intensity for arithmetic mean (a) and geometric mean (b) for duplicate Kato-Katz thick smears of a single stool sample. Black dots on crosses are the validation results from 34 locations in Uganda with observed prevalence above 10% selected from a survey with 146 schools and the dots are the simulated results. EPS, eggs-per-slide.

Comparison with simulation results is shown in Fig 3, plotting the estimated sensitivities for the validation data in relation to arithmetic and geometric mean infections as well as all the simulated populations for duplicate Kato-Katz thick smears from a single sample. For low infection-intensities the relations match reasonably well, starting off around 50% and increasing to 80% for 5 EPS arithmetic mean and 1.25 EPS geometric mean, respectively. The simulations underestimate the sensitivity for larger infection intensity by never surpassing 90% even for high infection intensity. This indicates that in the simulation there is a larger number of light infections compared to the validation datasets.

## Discussion

We estimated the ‘true’ prevalence of *S. mansoni* from population summary measures of Kato-Katz diagnostics for sampling schemes varying from a single slide to triplicate slides on 3 consecutive days. We considered two population summary measures of infection intensity; the arithmetic and geometric mean infection intensity for both the full population or only the positive individuals and observed prevalence. Our aim was to improve estimation of the ‘true’ prevalence from the basic approach of using a constant sensitivity in the absence of individual-level data.

We determined the infection intensity-dependent sensitivity for individuals by fitting a Bayesian egg count model for Kato-Katz to data from different transmission settings in sub-Saharan Africa. We have previously shown that it is possible to infer on the ‘true’ prevalence from individual-level data [8, 12]. There have been other approaches to determine ‘true’ prevalence of schistosomiasis. Prada et al. use a very similar approach to model Kato-Katz results but do not take into account the difference between day-to-day and slide-to-slide variation [13]. Clements et al. use latent class analysis on binary Kato-Katz results with conditional dependence introduced by fixed effects which can be a flexible model for binary tests but is not ideal for a situation where multiple diagnostic tests depend on the same latent variable, here infection intensity, and does not allow individual-level simulations [29]. Similarly, Lindholz et al. use latent class analysis to adjust for imperfect reference standards but with the assumption of conditional independence. Our approach allows for the direct inclusion of count data and models variations in egg output on the individual level. Therefore, we deem it the most appropriate for the simulation of individual level data to study the relation between prevalence and infection-intensity [30].

Heavy infections (≥ 400 EPG) are reliably detected even from a single stool sample, while for moderate infection intensities (100–399 EPG) two days are necessary to achieve more than 90% sensitivity. Very light infections below 10 EPG are difficult to detect even for multiple Kato-Katz thick smears performed over 3 consecutive days because the very low density of eggs makes them unlikely to show up in a thick smear of 41.7 mg. Thus, diagnostic sensitivity mostly affects light infections and because of the overdispersion of infection-intensities in the population and the highly non-linear relation between infection-intensity and sensitivity the overall sensitivity is never as low as for light infections. An infection with a single *S. mansoni* worm-pair has been estimated to produce between 1 and 5 EPG; a range where the sensitivity of Kato-Katz is strongly dependent on the infection intensity [31]. For example, for three stool samples with two slides each, sensitivity of Kato-Katz is 20% at 1 EPG but 50% at 4 EPG. Therefore, the number of samples needed to detect even infection with a single worm-pair cannot easily be inferred.

Simulating populations was done using the assumptions of a negative binomial distribution of worms and monogamous mating [24, 26, 27]. Geometric and arithmetic means were calculated for both, the total population and the infected individuals only, as it was expected that the infection intensity of infected people could contain valuable information about the sensitivity. However, estimating means from only the positives in sample sizes of 30 or 50 individuals leads to large uncertainty rendering this measure impractical. Our results showed the strong dependence of the sensitivity on the infection intensity confirming once more that a constant sensitivity is not sufficient to estimate ‘true’ prevalence. The assumption of negative binomial distribution of worms in a population links the prevalence of the disease to the mean worm burden. Furthermore the relation between the observed and ‘true’ prevalence is rather linear.

The statistical models used to describe the relation between sensitivity and infection intensity are based on the assumption that the sensitivity is monotonically increasing with infection-intensity and reaches 100% for heavy infections. We also assumed that the relation between the observed and the ‘true’ prevalence is monotonically increasing and at 100% observed prevalence the ‘true’ prevalence is 100% too. Estimating not just the mean but a whole distribution about the sensitivity enabled us to quantify the uncertainty of the sensitivity and therefore obtain prior distributions for Bayesian models including the sensitivity as a parameter. Translation of the observed prevalence to the ‘true’ one can be done by hand using the parameters given in the results section and provided in supplementary material or an excel spreadsheet can be created to facilitate application by disease control managers. De Vlas et al. (1997) developed and validated a chart to translate Kato-Katz data from a single stool sample to ‘true’ prevalence [32]. Here we present parametric functions for six different sampling schemes to estimate both sensitivity and ‘true’ prevalence from arithmetic and geometric mean infection intensities.

The validation data agrees with the simulated data for lower infection intensities. However, sensitivity saturates around 90% in the simulated data, while fitting a model to individual-level data estimates a sensitivity of close to 100% being reached. This indicates that either the negative binomial distribution does not accurately capture the distribution of worms at high infection intensities but over-estimates the number of light infections or the gamma distribution under-estimates the number of light infections. The former could imply that at high transmission intensity, exposed individuals harbor a large number of worms, while some people are still not exposed at all leading to fewer light infections than expected under the negative binomial assumption. An improvement of our model could be to vary the worm aggregation parameter with infection intensity.

The uncertainty about the number of light infections of *S. mansoni* is important in the context of disease control and particularly elimination. Discussions about diagnostic specificity of the point-of-care circulating cathodic antigen (POC-CCA) diagnostic technique include a similar argument when considering whether the additional positives by POC-CCA are ‘true’ infections or false-positives [9]. Haggag et al. (2019) found that a large number of POC-CCA positives but Kato-Katz negatives shows no egg excretion even when samples are taken over 30 days [10]. Possible explanations include that that the number of very light infections might be over-estimated but also juvenile or single worm infections caused by the sex-bias and lack of efficacy for juvenile worms with the antischistosomal drug praziquantel.

### Conclusion

Taken together, we showed that there is important variation in the population-level sensitivity of Kato-Katz, i.e., the ratio between observed and ‘true’ prevalence, with mean infection intensity. We confirmed that the relation between the sensitivity and the infection intensity can be simulated using our egg count model for Kato-Katz observations combined with standard assumptions for the worm distribution in a population. Our parametric model fitted on simulated data can be used to translate observed prevalence into ‘true’ prevalence when either the arithmetic or geometric mean infection intensity are available. Moreover our results provide Bayesian priors when modeling historical survey data aggregated at the population level.

## Supporting information

S1 Fig Schematic of the model structure. Data are in black boxes, models in blue boxes, model estimates in grey boxes, model fitting procedures in orange boxes, the simulation in a green box, and prediction and validation in a violet box. The comparison of interest is denoted by a red arrow.
(PNG)

S2 Fig Infection intensity-dependent sensitivity of Kato-Katz for intensities up to 200 EPG and five different sampling schemes. d denotes the number of days where stool samples were collected, and s the number of slides per stool sample.
(TIF)

S3 Fig Infection intensity-dependent sensitivity of Kato-Katz for intensities up to 25 EPG for five different sampling schemes. The number before the d denotes the number of days where stool samples were collected, and the one before s the number of slides per stool sample. The vertical line denotes an estimate of the infection intensity of an infection with a single worm-pair.
(TIF)

S4 Fig Overall sensitivity of Kato-Katz in relation to geometric and arithmetic mean of only positive individuals for six different sampling schemes in simulations with 50 individuals per location. The sampling scheme is denoted on the right side with the first number referring to the number of stool samples and the second to the number of slides per stool sample. Observed prevalence is shown in color and EPS refers to eggs-per-slide.
(PNG)

S5 Fig Estimated relation between geometric mean infection intensity of the total population and sensitivity. Posterior mean and 95% BCI as red line and shading for each sampling scheme and a sample size of 50. The black dots indicate the simulated data and EPG refers to eggs-per-gram.
(TIF)

S6 Fig Estimated relation between arithmetic mean infection intensity of the total population and sensitivity. Posterior mean and 95% BCI as red line and shading for each sampling scheme and a sample size of 50. The black dots indicate the simulated data and EPG refers to eggs-per-gram.
(PNG)

S7 Fig Estimated relation between observed prevalence and ‘true’ prevalence. Posterior mean and 95% BCI as red line and shading for each sampling scheme and a sample size of 50. The black dots indicate the simulated data and EPG refers to eggs-per-gram.
(PNG)

S1 Table Validation dataset. The data contain Kato-Katz thick smear egg-counts from two slides on one sample of 1,769 individuals from 34 schools in Uganda.
(CSV)

S2 Table Posterior estimates (mean and 95% BCI) of the parameters describing the relation between arithmetic mean of the total population and sensitivity for six sampling schemes and two samples. (PDF)

S3 Table Posterior estimates (mean and 95% BCI) of the parameters describing the relation between observed prevalence and ‘true’ prevalence for six sampling schemes and two samples. (PDF)

S1 File Stan code of egg-count model. Stan code of the model used to fit the egg-count model.
(TXT)

S2 File R code of egg-count simulation. R code of the simulation model used to create individual-level data.
(TXT)

S3 File Stan code of statistical model fit to geometric mean simulation. (TXT)

S4 File Stan code of statistical model fit to arithmetic mean simulation. (TXT)

S5 File Stan code of statistical model fit to observed prevalence simulation. (TXT)

S6 File Stan code of egg-count model used for validation including informative priors. (TXT)
