# Supplementary figures and images for: Estimating true prevalence of Schistosoma mansoni from population summary measures based on the Kato-Katz diagnostic technique
Source: PLoS Negl Trop Dis. 2021 Apr 5;15(4):e0009310. doi: 10.1371/journal.pntd.0009310 (PMC8062092; doi:10.1371/journal.pntd.0009310)

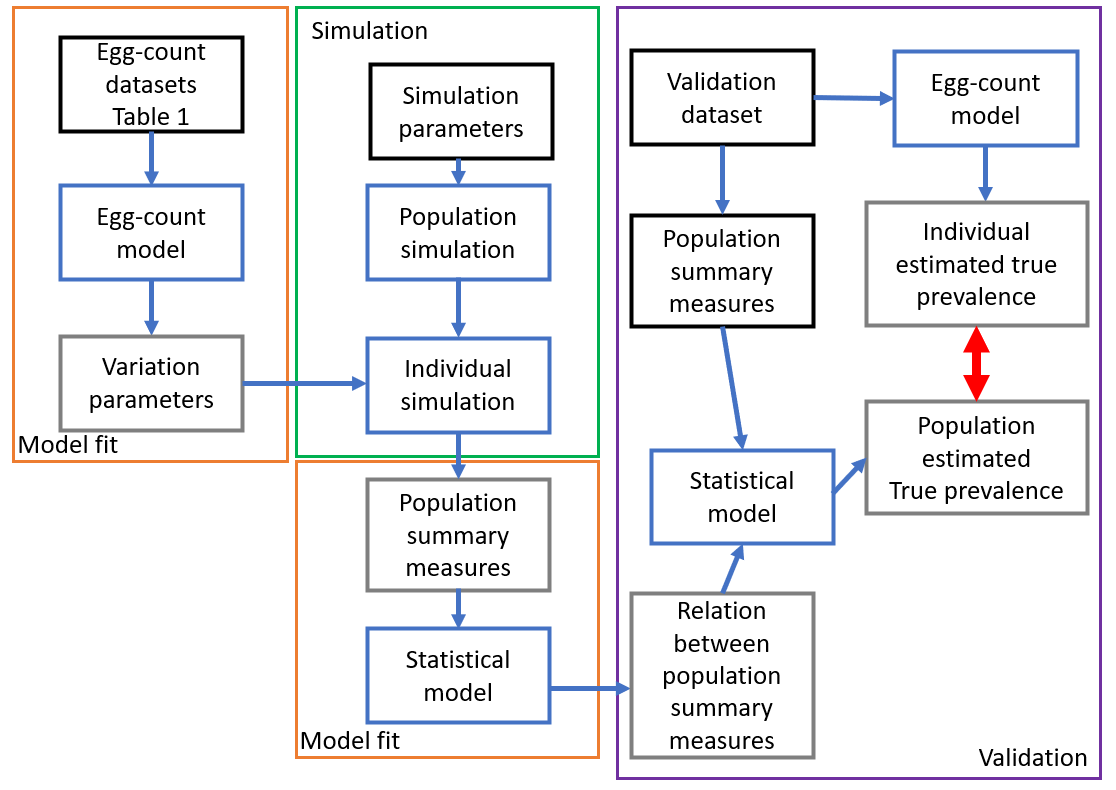

Supplement: S1 Fig — Data are in black boxes, models in blue boxes, model estimates in grey boxes, model fitting procedures in orange boxes, the simulation in a green box, and prediction and validation in a violet box. The comparison of interest is denoted by a red arrow. (PNG) [file pntd.0009310.s001.png]

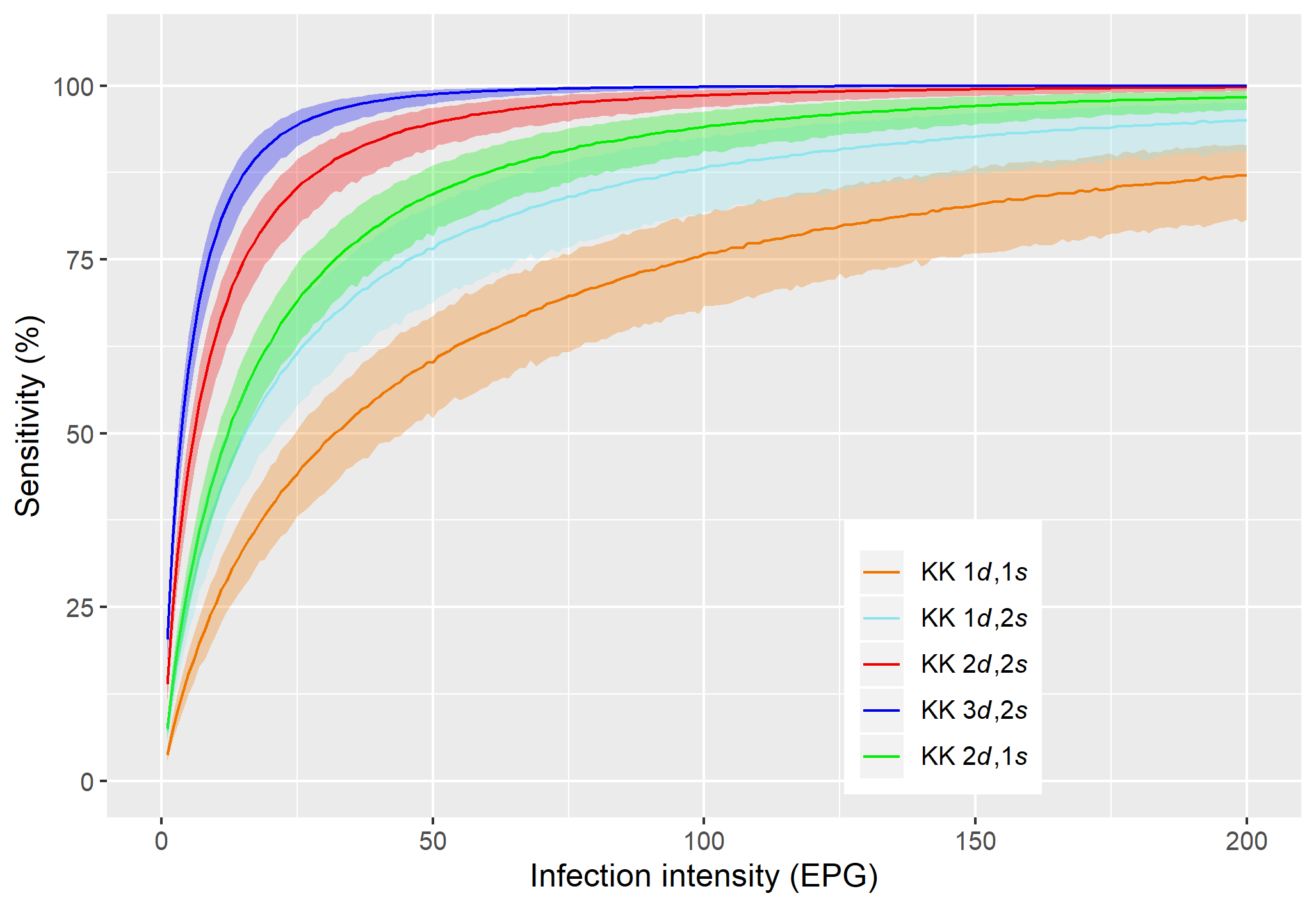

Supplement: S2 Fig — d denotes the number of days where stool samples were collected, and s the number of slides per stool sample. (TIF) [file pntd.0009310.s002.tif]

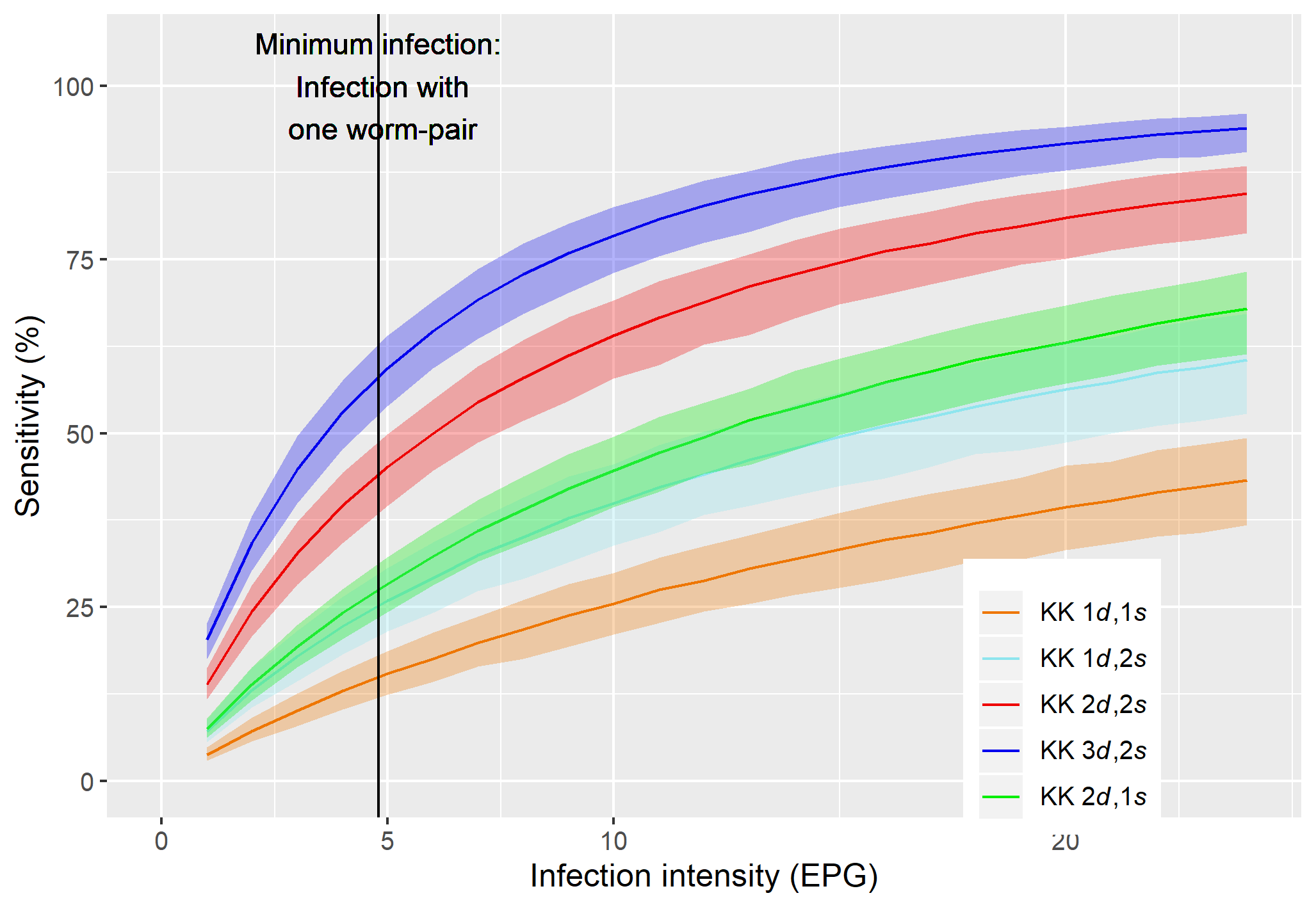

Supplement: S3 Fig — The number before the d denotes the number of days where stool samples were collected, and the one before s the number of slides per stool sample. The vertical line denotes an estimate of the infection intensity of an infection with a single worm-pair. (TIF) [file pntd.0009310.s003.tif]

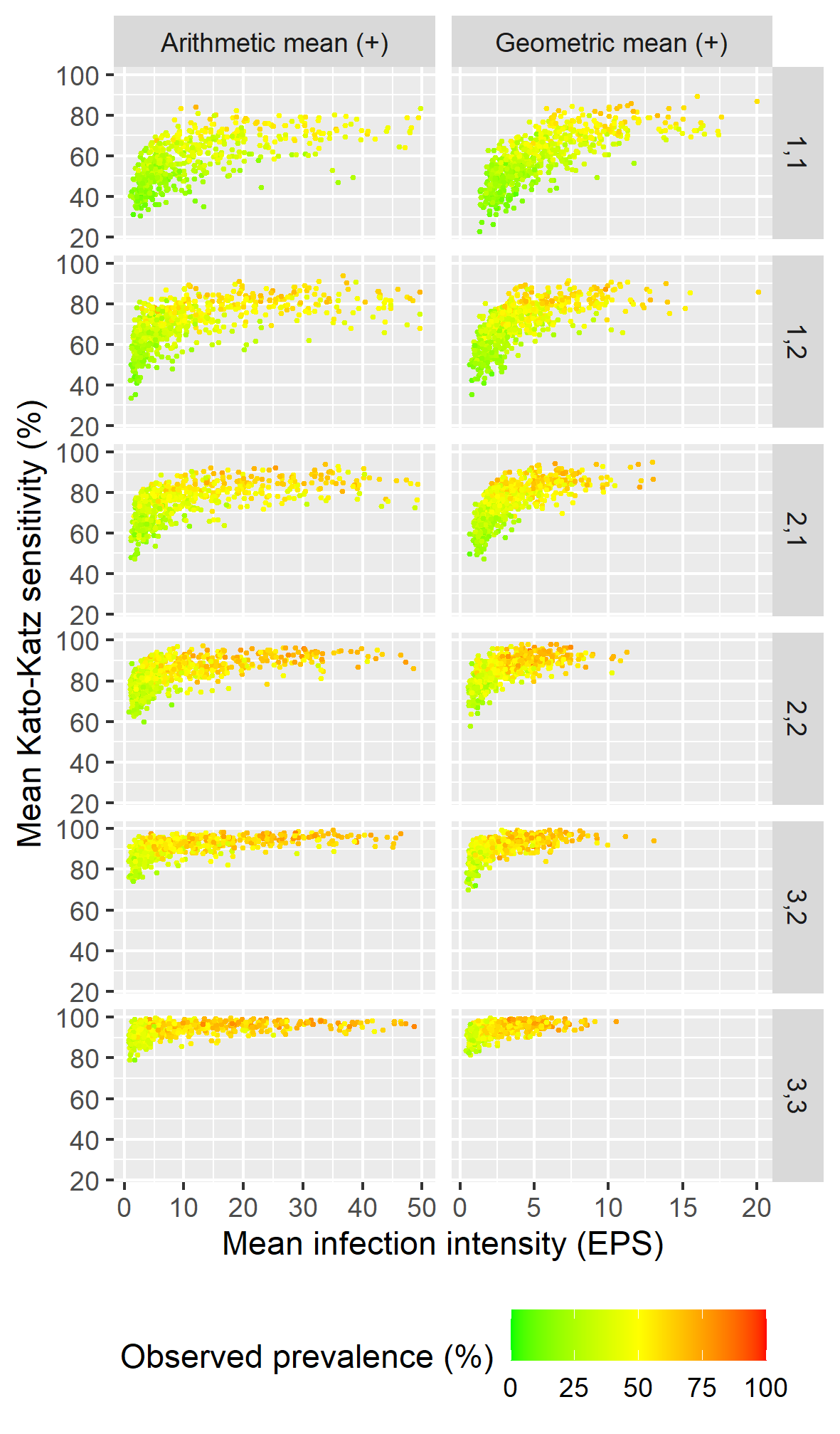

Supplement: S4 Fig — The sampling scheme is denoted on the right side with the first number referring to the number of stool samples and the second to the number of slides per stool sample. Observed prevalence is shown in color and EPS refers to eggs-per-slide. (PNG) [file pntd.0009310.s004.png]

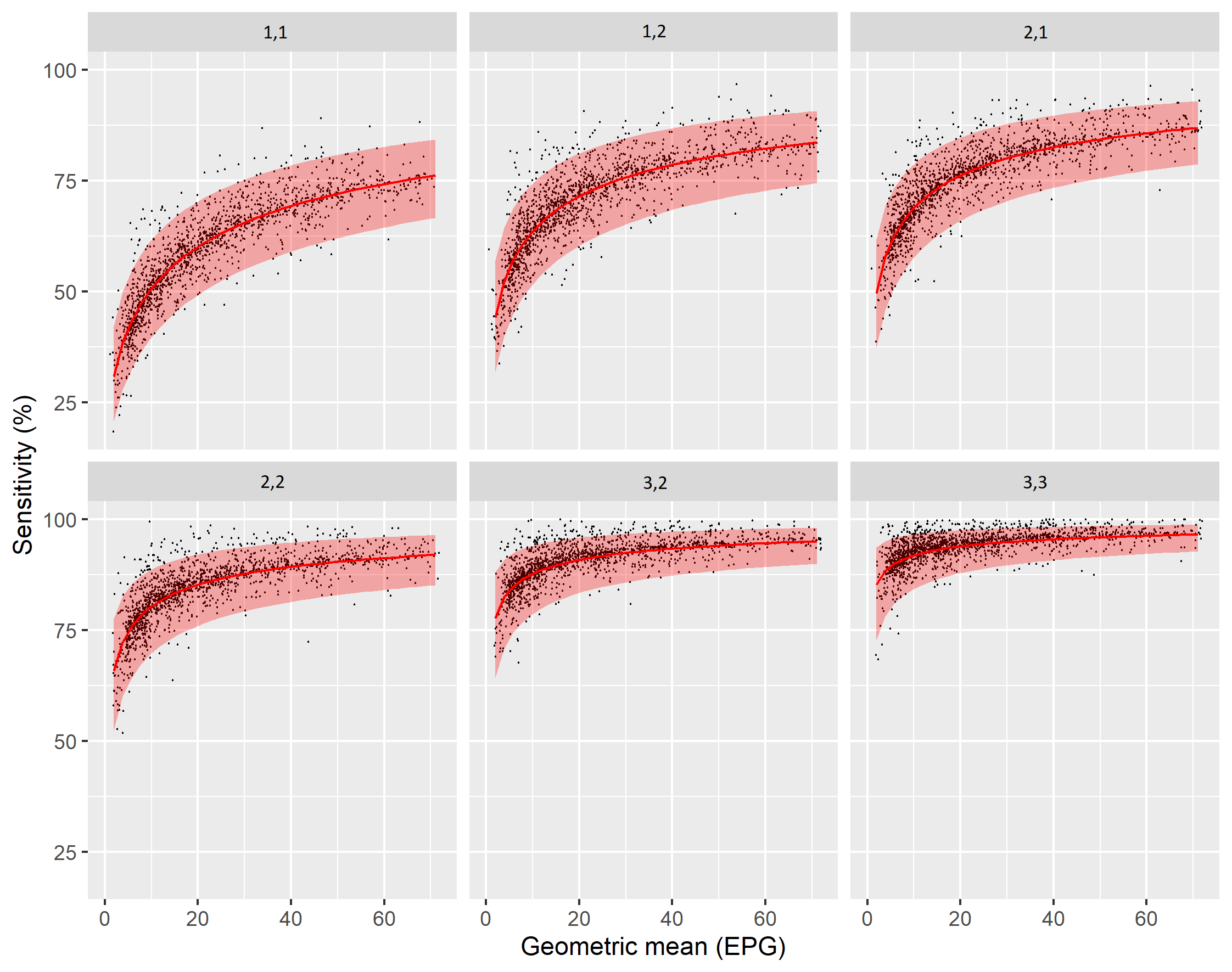

Supplement: S5 Fig — Posterior mean and 95% BCI as red line and shading for each sampling scheme and a sample size of 50. The black dots indicate the simulated data and EPG refers to eggs-per-gram. (TIF) [file pntd.0009310.s005.tif]

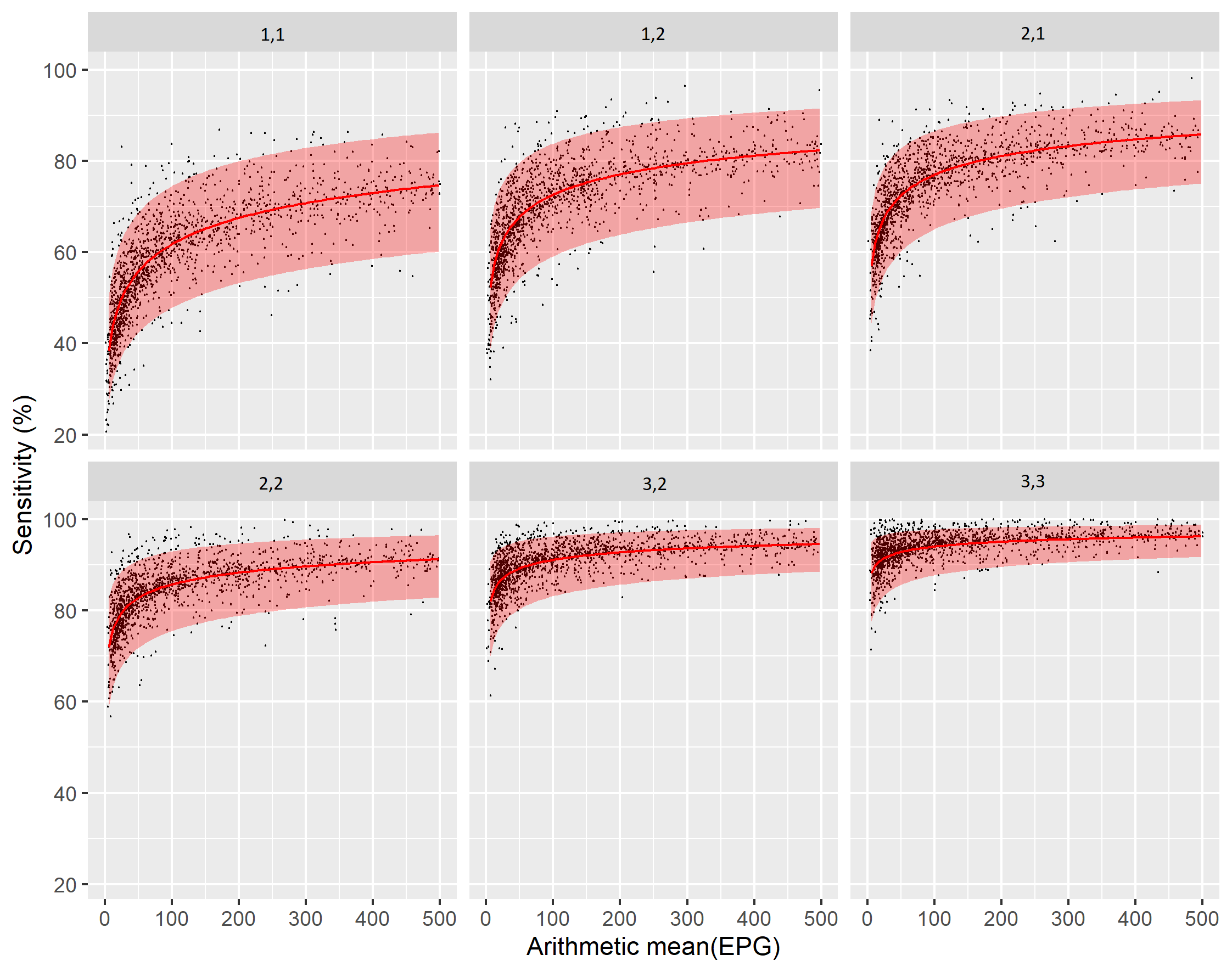

Supplement: S6 Fig — Posterior mean and 95% BCI as red line and shading for each sampling scheme and a sample size of 50. The black dots indicate the simulated data and EPG refers to eggs-per-gram. (PNG) [file pntd.0009310.s006.png]

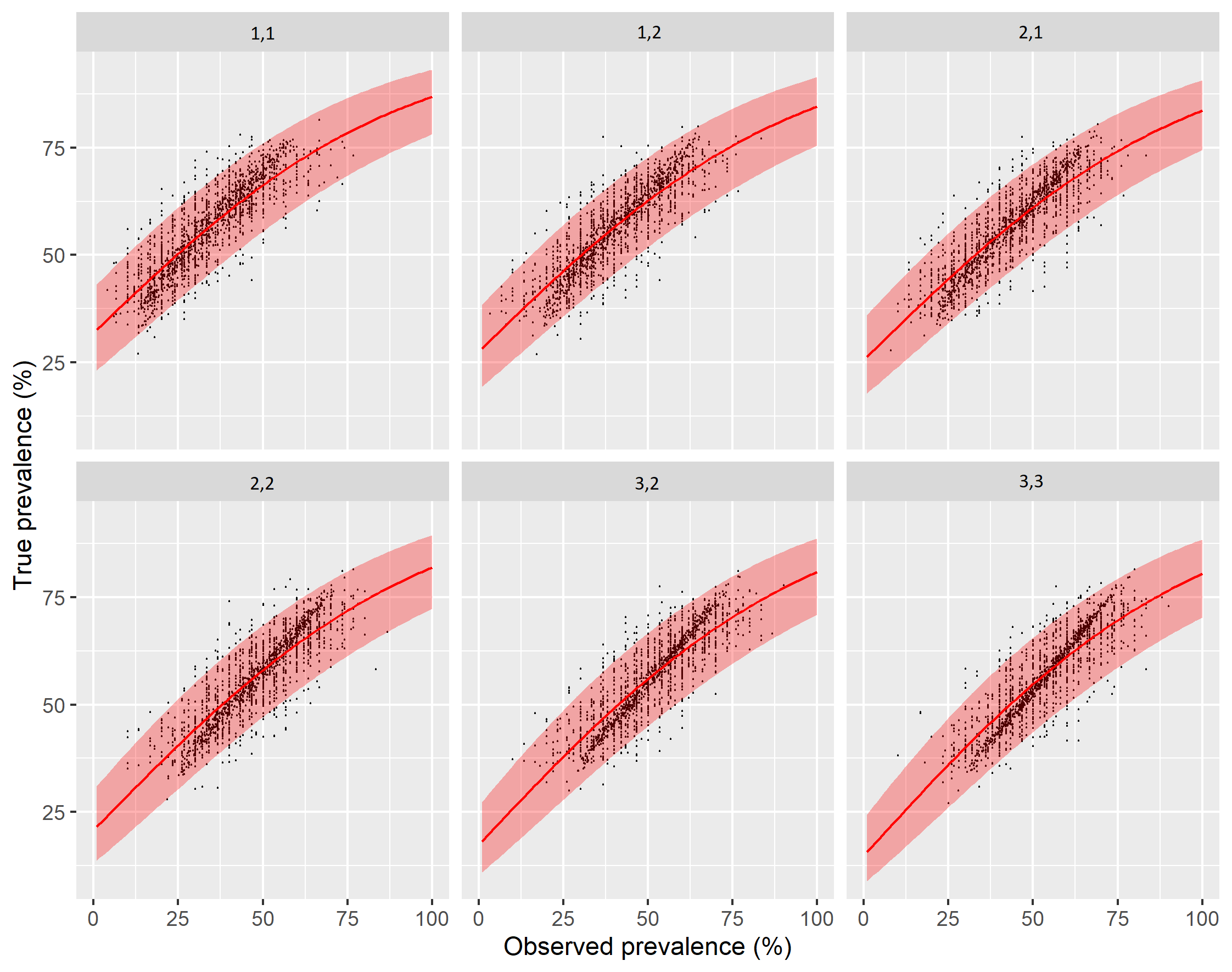

Supplement: S7 Fig — Posterior mean and 95% BCI as red line and shading for each sampling scheme and a sample size of 50. The black dots indicate the simulated data and EPG refers to eggs-per-gram. (PNG) [file pntd.0009310.s007.png]
