# Supplementary material for: Estimating true prevalence of Schistosoma mansoni from population summary measures based on the Kato-Katz diagnostic technique
Source: PLoS Negl Trop Dis. 2021 Apr 5;15(4):e0009310. doi: 10.1371/journal.pntd.0009310 (PMC8062092; doi:10.1371/journal.pntd.0009310)
Supplement: S2 Table — (PDF) [file pntd.0009310.s009.pdf]

S1 Table: Posterior estimates (mean and 95% BCI) of the parameters describing the relation between arithmetic mean of the total population and sensitivity for six sampling schemes and two sample.

**Scheme**	a_0	a_1	a_2	b_0	b_1
**N=50**					
1d1s	-7.81 (-9.89 - -5.98)	8.94 (7.12 - 11.02)	23.39 (18.22 - 29.35)	-3.71 (-3.8 - -3.61)	0.11 (0.07 - 0.15)
1d2s	-6.23 (-8.32 - -4.46)	7.81 (6.03 - 9.9)	21.84 (16.45 - 28.1)	-3.82 (-3.92 - -3.73)	0.04 (0 - 0.08)
2d1s	-6.41 (-8.76 - -4.56)	8.24 (6.39 - 10.58)	22.05 (16.69 - 28.99)	-4.01 (-4.11 - -3.91)	0.02 (-0.02 - 0.06)
2d2s	-4.51 (-6.46 - -2.9)	6.87 (5.26 - 8.81)	19.79 (14.68 - 25.97)	-4.2 (-4.3 - -4.1)	-0.05 (-0.1 - -0.01)
3d2s	-3.28 (-5.15 - -1.73)	6.14 (4.59 - 8)	18.31 (13.13 - 24.5)	-4.5 (-4.6 - -4.41)	-0.12 (-0.17 - -0.08)
3d3s	-2.28 (-4.14 - -0.76)	5.51 (3.99 - 7.37)	17.7 (12.3 - 24.29)	-4.78 (-4.88 - -4.68)	-0.18 (-0.22 - -0.14)
**N=30**					
1d1s	-7.96 (-10.14 - -6.12)	9.12 (7.29 - 11.3)	23.92 (18.65 - 30.15)	-3.32 (-3.42 - -3.23)	0.11 (0.07 - 0.14)
1d2s	-6.33 (-8.32 - -4.65)	7.94 (6.26 - 9.92)	22.15 (16.99 - 28.25)	-3.37 (-3.47 - -3.28)	0.04 (0 - 0.07)
2d1s	-5.88 (-7.89 - -4.24)	7.74 (6.11 - 9.76)	20.46 (15.67 - 26.34)	-3.57 (-3.66 - -3.47)	0.02 (-0.02 - 0.06)
2d2s	-4.08 (-5.98 - -2.48)	6.46 (4.86 - 8.35)	18.5 (13.37 - 24.61)	-3.7 (-3.8 - -3.61)	-0.05 (-0.09 - -0.01)
3d2s	-3.11 (-5.03 - -1.53)	5.95 (4.38 - 7.89)	18.28 (12.93 - 24.77)	-4 (-4.1 - -3.91)	-0.11 (-0.15 - -0.07)
3d3s	-2.1 (-3.83 - -0.67)	5.31 (3.89 - 7.04)	17.71 (12.32 - 24.15)	-4.29 (-4.38 - -4.19)	-0.17 (-0.21 - -0.13)

The mean estimate for the 'true' prevalence is calculated by $p = p^{obs} / \operatorname{logit}^{-1}(a_0 + a_1 \cdot (\mu_a/25)^{1/a_2})$ when the arithmetic mean infection intensity μ_a in eggs-per-slide (EPS) is known.
The probability distribution of the 'true' prevalence is given by Beta(α,β), where $\alpha = p/\nu$, $\beta = (1-p)/\nu$, and $\nu = \exp(b_0 + b_1 \log(\mu_a/25))$.
