# Supplementary material for: Estimating true prevalence of Schistosoma mansoni from population summary measures based on the Kato-Katz diagnostic technique
Source: PLoS Negl Trop Dis. 2021 Apr 5;15(4):e0009310. doi: 10.1371/journal.pntd.0009310 (PMC8062092; doi:10.1371/journal.pntd.0009310)
Supplement: S3 Table — (PDF) [file pntd.0009310.s010.pdf]

Table S2: Posterior estimates (mean and 95% BCI) of the parameters describing the relation between observed prevalence and 'true' prevalence for six sampling schemes and two sample.

**Scheme**	a_0	a_1	b_0	b_1
**N=50**				
1d1s	0.32 (0.31 - 0.33)	2.21 (2.17 - 2.25)	0.01 (0.01 - 0.01)	0 (0 - 0)
1d2s	0.28 (0.27 - 0.28)	2.11 (2.07 - 2.14)	0.01 (0.01 - 0.01)	0 (0 - 0)
2d1s	0.26 (0.25 - 0.26)	2.07 (2.03 - 2.11)	0.01 (0.01 - 0.01)	0 (0 - 0)
2d2s	0.21 (0.2 - 0.22)	2.03 (1.99 - 2.07)	0.01 (0.01 - 0.01)	0 (0 - 0)
3d2s	0.18 (0.16 - 0.19)	2.02 (1.98 - 2.05)	0.01 (0.01 - 0.01)	0 (0 - 0.01)
3d3s	0.15 (0.14 - 0.16)	2.02 (1.98 - 2.06)	0.01 (0.01 - 0.01)	0 (0 - 0.01)
**N=30**				
1d1s	0.36 (0.35 - 0.36)	1.98 (1.93 - 2.02)	0.02 (0.02 - 0.02)	0 (0 - 0)
1d2s	0.32 (0.31 - 0.33)	1.88 (1.84 - 1.92)	0.02 (0.01 - 0.02)	0 (0 - 0)
2d1s	0.31 (0.3 - 0.31)	1.84 (1.8 - 1.88)	0.02 (0.01 - 0.02)	0 (0 - 0)
2d2s	0.27 (0.26 - 0.28)	1.8 (1.75 - 1.84)	0.02 (0.02 - 0.02)	0 (0 - 0)
3d2s	0.24 (0.23 - 0.25)	1.79 (1.75 - 1.83)	0.02 (0.02 - 0.02)	0 (0 - 0.01)
3d3s	0.23 (0.21 - 0.24)	1.79 (1.74 - 1.83)	0.02 (0.02 - 0.02)	0 (0 - 0.01)

The mean estimate for the 'true' prevalence is calculated by $p = (2\,\mathrm{logit}^{-1}(a_1 p_{obs}) - 1)(1 - a_0) + a_0$
The probability distribution of the 'true' prevalence is given by Beta(α,β), where $\alpha = p/\nu$, $\beta = (1-p)/\nu$, and $\nu = b_0 + b_1 p_{obs}$.
